# The Pharmacokinetics of Vitamin C

**DOI:** 10.3390/nu11102412

**Published:** 2019-10-09

**Authors:** Jens Lykkesfeldt, Pernille Tveden-Nyborg

**Affiliations:** Faculty of Health and Medical Sciences, Department of Veterinary and Animal Sciences, University of Copenhagen, DK-1870 Frederiksberg C, Denmark; ptn@sund.ku.dk

**Keywords:** vitamin C, pharmacokinetics, homeostasis, human disease

## Abstract

The pharmacokinetics of vitamin C (vitC) is indeed complex. Regulated primarily by a family of saturable sodium dependent vitC transporters (SVCTs), the absorption and elimination are highly dose-dependent. Moreover, the tissue specific expression levels and subtypes of these SVCTs result in a compartmentalized distribution pattern with a diverse range of organ concentrations of vitC at homeostasis ranging from about 0.2 mM in the muscle and heart, and up to 10 mM in the brain and adrenal gland. The homeostasis of vitC is influenced by several factors, including genetic polymorphisms and environmental and lifestyle factors such as smoking and diet, as well as diseases. Going from physiological to pharmacological doses, vitC pharmacokinetics change from zero to first order, rendering the precise calculation of dosing regimens in, for example, cancer and sepsis treatment possible. Unfortunately, the complex pharmacokinetics of vitC has often been overlooked in the design of intervention studies, giving rise to misinterpretations and erroneous conclusions. The present review outlines the diverse aspects of vitC pharmacokinetics and examines how they affect vitC homeostasis under a variety of conditions.

## 1. Introduction

Humans rely solely on dietary intake for the maintenance of the body pool of vitamin C (vitC). In contrast to the vast majority of vertebrates, in which l-gulonolactone oxidase catalyzes the final step in the biosynthesis of ascorbic acid, evolutionally conserved deletions have made the corresponding gene inactive in primates, flying mammals, guinea pigs, and some bird and fish species, thereby disabling its formation [1]. This evolutionary event may in fact have resulted in an adaptational process where our ability to prevent vitC deficiency has been improved by various measures changing the pharmacokinetics, including more efficient absorption, recycling, and renal reuptake of vitC compared to vitC synthesizing species [2,3]. 

The absorption, distribution, metabolism, and excretion of vitC in humans is highly complex and unlike that of most low molecular weight compounds. The majority of intestinal uptake, tissue distribution, and renal reuptake is handled by the sodium-dependent vitC transporter (SVCT) family of proteins [4] that cotransports sodium ions and ascorbate (ASC) across membranes with the ability to generate considerable concentration gradients [5,6]. It is the differential expression, substrate affinity, and concentration dependency of the SVCTs between organs that gives rise to the unique compartmentalization and nonlinear pharmacokinetics of vitC at physiological levels [7]. 

The hydrophilic nature of ASC and the likely resulting absence of passive diffusion across biological membranes has puzzled pharmacologists. However, two decades ago, active transport of vitC was found to be essential for life, when Sotiriou et al. showed that SVCT2 knockout mice die immediately after birth from respiratory failure with severe brain hemorrhage [8]. Acknowledging the role of SVCTs in vitC homeostasis has naturally sparked an interest in possible differences in SVCT activity between individuals and potential impact on vitC status. Thus, a number of polymorphisms have been identified, and these may affect the pharmacokinetics of vitC significantly. Although not investigated in clinical studies yet, pharmacokinetic modelling has suggested that several of the identified SVCT alleles result in a lower plasma steady state level and consequently completely altered homeostasis [9] with the lowest saturation level leading to permanent vitC deficiency, i.e., a plasma concentration < 23 µM [10,11]. 

In contrast to the physiological concentrations achievable by oral ingestion, pharmacological concentrations, i.e., millimolar plasma concentrations, can be reached by parenteral administration, mostly intravenous infusion [12]. Interestingly, the pharmacokinetics of vitC appears to change from zero to first order following high-dose infusion displaying a constant and dose-independent half-life [13]. 

A final factor contributing to the complexity of vitC pharmacology is its metabolism. Low molecular weight drugs and xenobiotics are normally metabolized by a combination of phase I and II enzymes leading to oxidized and conjugated metabolites with increased water solubility and enhanced clearance. VitC takes part in numerous physiological reactions as an electron donor [14]. Acting both as a specific cofactor or antioxidant, ASC is oxidized to the ascorbyl radical, which subsequently may undergo dismutation to form ASC and dehydroascorbic acid (DHA) [15]. Although DHA has a half-life of only a few minutes [16], it is normally reduced back to ASC by enzymatic means, an intracellular process that is both efficient and quantitative in healthy individuals. However, it has been shown that the recycling process may be inadequate during disease and among smokers, for example, resulting in an increased turnover of vitC [17,18]. Thus, increased intake of vitC may be necessary to achieve homeostasis in high-risk individuals.

The present review outlines the pharmacokinetics of vitC under various conditions and discusses how it affects the vitC status.

## 2. Pharmacokinetics of Vitamin C 

Pharmacokinetics constitutes the description of absorption, distribution, metabolism, and excretion of drugs. Pharmacokinetics is based on a number of theoretical models, all of which have a set of assumptions that need to be fulfilled for their validity. Compared to a typical orally administered low molecular weight drug, vitC differs in multiple ways with respect to pharmacokinetic properties [19]. Unfortunately, lack of proper attention to particularly the nonlinearity of vitC pharmacokinetics has led to misinterpretation of a major part of the clinical literature as reviewed elsewhere [11,19,20]. In the following, the kinetics of vitC is explored in more detail.

### 2.1. Oral Route of Administration

Oral ingestion of food or supplements is the primary route of administration for vitC. VitC is ubiquitous in nature and particularly fruits and vegetables contain relatively large amounts of ASC [21]. For healthy individuals, it is possible to get sufficient amounts of vitC through the diet provided it contains high amounts of vitC-rich sources [22,23]. However, in many diseases and in people with very poor vitC status including smokers, for example, the dietary intake may be insufficient to provide adequate amounts of vitC [19,24,25].

#### 2.1.1. Absorption

VitC exists primarily in two forms in vivo, ASC (reduced form) and DHA (oxidized form), of which the former is by far the predominant [26]. Due to the efficient intracellular recycling of DHA to ASC by most cell types, the total available vitC capacity is considered the combined pool of ASC and DHA [27]. With regard to vitC, three potential modes of membrane transport exist: passive diffusion, facilitated diffusion, and active transport [6]. 

For most low molecular weight drugs, simple diffusion is the primary means of membrane transport. However, vitC is predominantly represented by its anionic form (>99.9%) at neutral pH and is highly water-soluble. As such, it will only be able to diffuse across the plasma membrane at a relatively slow rate even in the presence of a considerable concentration gradient. However, in the milieu of the stomach (pH 1) or small intestine (pH 5), the proportion of unionized ascorbic acid increases to 99.9% and 15%, respectively, and under these local conditions, passive diffusion could perhaps play a more significant role in vitC uptake. Studies in individuals with normal vitC status have reported similar times to maximal plasma concentration following oral administration of ascorbic and erythorbic acid, respectively [28,29], even though erythorbic acid, an isoform of ASC with low vitC activity, is poorly transported by epithelial SVCT1 [30]. However, it remains undisclosed if passive diffusion of ASC contributes significantly to its absorption from these compartments. 

Facilitated diffusion across membranes occurs through carrier proteins but like passive diffusion, it depends on an electrochemical gradient. DHA has been shown to compete with glucose for transport through several glucose transporters [31,32]. While only present in negligible amounts in the blood of healthy individuals [17,33], intestinal concentrations are presumably much higher, most likely due to the absence of intracellular recycling and relatively higher concentration in foodstuffs. This may explain the repeated finding of similar bioavailability of ASC and DHA as vitC sources [2,34,35,36]. Moreover, this could explain the observation of equal absorption rates of ascorbic and erythorbic acid from the intestine as dehydroerythorbic acid would be expected to pass through glucose transporters. DHA uptake is expectedly inhibited by excess glucose, while the maximal rates of uptake for ASC and DHA are similar when glucose is absent [37].

**Figure 1:**
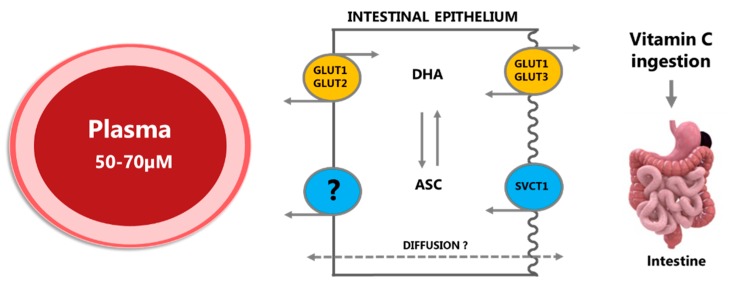
Ingested vitamin C (vitC) is absorbed across the intestinal epithelium primarily by membrane transporters in the apical brush border membrane, either as ascorbate (ASC) by sodium-coupled active transport via the SVCT1 transporter or as dehydroascorbic acid (DHA) through facilitated diffusion via GLUT1 or GLUT3 transporters. Once inside the cell, DHA is efficiently converted to ASC or transported to the blood stream by GLUT1 and GLUT2 in the basolateral membrane, hereby maintaining a low intracellular concentration and facilitating further DHA uptake. ASC is conveyed to plasma by diffusion, possibly also by facilitated diffusion through volume-sensitive anion channels or by yet unidentified active transporters; the precise efflux mechanisms remain unknown. Modified from [5].

Finally, concentration gradient-independent active transport plays a significant role in vitC absorption. As early as the 1970s, it was observed that the bioavailability of ASC is highly dose-dependent [38]. Increasing oral doses were shown to lead to decreasing absorption fractions and it was concluded by several authors that intestinal ASC absorption is subject to saturable active transport [38,39]. Malo and Wilson discovered that DHA and ASC are taken up by separate mechanisms in the intestine and that uptake of ASC is sodium-dependent [37]. This coincided with the discovery and characterization of the SVCT family of transporters by Tsukaguchi et al. [4]. They subsequently showed that the intestine contains the low affinity/high capacity active transporter SVCT1 [30]. Thus, ASC is efficiently transported across the apical membrane of the intestinal epithelial cells via active transport but its release into the blood stream is less well understood. As intracellular vitC is effectively kept reduced, facilitating further uptake of DHA, efflux to the blood through glucose transporters is unlikely to provide a significant contribution. As mentioned above, the intracellular pH of 7.0 renders the anionic ASC predominant (99.9%) and given its hydrophilic nature, passive efflux of ascorbic acid via simple diffusion will be relatively slow. However, as the cellular release of vitC to the blood stream is vital for the absorption process and must occur to a high extend considering the rapid uptake of vitC (plasma Tmax of about 3 h [29]), it strongly implies the existence of yet undiscovered channels or transporters facilitating vitC efflux. It has been proposed that ASC efflux may occur through volume-sensitive anion channels in the basolateral membranes of epithelial cells [6]. In the brain, however, studies in human microvascular pericytes have shown that volume-sensitive anion channels are apparently not involved in the ASC efflux from these cells and may therefore not represent a general mechanism of basal ASC efflux [40]. A schematic overview of intestinal vitC absorption is shown in Figure 1.

#### 2.1.2. Distribution

**Figure 2:**
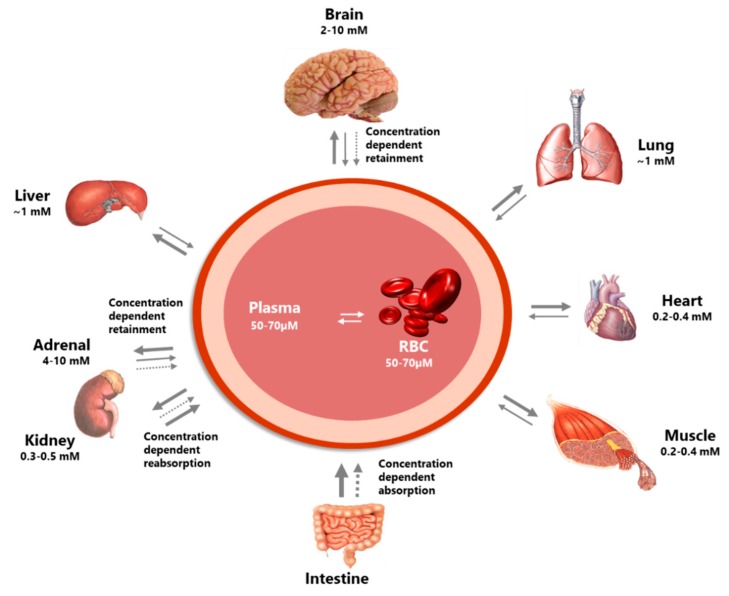
The figure illustrates the highly differential distribution of vitC in the body. Several organs have concentration-dependent mechanisms for the retention of vitC, maintaining high levels during times of inadequate supply at the expense of other organs. Particularly protected is the brain. In addition, the concentration-dependent absorption and re-absorption mechanisms contribute to the homeostatic control of the vitC in the body. Modified from [5].

The distribution of vitC is highly compartmentalized (Figure 2). Simple diffusion is unlikely to play a major role in vitC transport across membranes, at least in the further distribution from the blood stream. From a theoretical point of view, ASC plasma steady state concentrations would be 2.5-fold higher than in tissue as calculated by a dissociation-determined equilibrium. In reality, intracellular concentrations of ASC range from about 0.5 to 10 mM compared to the mere 50–80 µM in the plasma of healthy individuals [7], confirming a many-fold preference for tissue. Although the glucose transporters (GLUTs 1–4 and 8) capable of facilitating diffusion of DHA are widely represented throughout the body [31,32,41,42,43], the negligible amount of oxidized vitC present in plasma of healthy individuals precludes that GLUT mediated transport per se is of major importance in the diverse distribution of vitC. One apparent exception is erythrocytes that do not contain SVCTs but are only able to take up vitC through facilitated diffusion [44,45,46]. Human erythrocytes are able to recycle DHA to ASC and maintain an intracellular vitC concentration similar to that of plasma [18]. It has been estimated that the erythrocytes alone are capable of reducing the total amount of vitC present in blood approximately once every 3 min [47,48]. Consequently, the recycling capacity of the erythrocytes may constitute a substantial antioxidant reserve in vivo. Recent investigations actually suggest that ASC is necessary for the structural integrity of the erythrocytes and that intracellular erythrocyte ASC is essential to maintain ASC plasma concentrations in vivo [49,50]. However, collectively speaking and considering the quantitative importance of mechanisms, ASC is primarily distributed via active transport. 

In contrast to epithelial ASC uptake and reuptake mediated by the high capacity/low affinity SVCT1 (Vmax of about 15 pmol/min/cell and Km of about 65–252 µM [5,30,51]), distribution from the blood stream to the various tissues is mainly governed by the slightly larger SVCT2 [52]. SVCT2 is a low capacity/high affinity transporter of vitC (Vmax of about 1 pmol/min/cell and Km of about 8–69 µM [5,30,51]) and is widely expressed in all organs [4]. The respective transport capacities and affinities for vitC fit well with the accepted notion that SVCT1 mediates the systemic vitC homeostasis, while SVCT2 secures local demands [53]. This is particularly evident for the brain, which upholds one of the highest concentrations of vitC in the body [7,54]. Transport of vitC into the brain is believed to take place through SVCT2s located in the choroid plexus [55], although it has been suggested that other yet undiscovered mechanisms may also be involved [56,57]. However, the pivotal role of SVCT2 in the brain remains undisputed as supported by convincing studies in *Slc23a2* knockout mice that display severe brain hemorrhage and high perinatal mortality [8].

**Figure 3:**
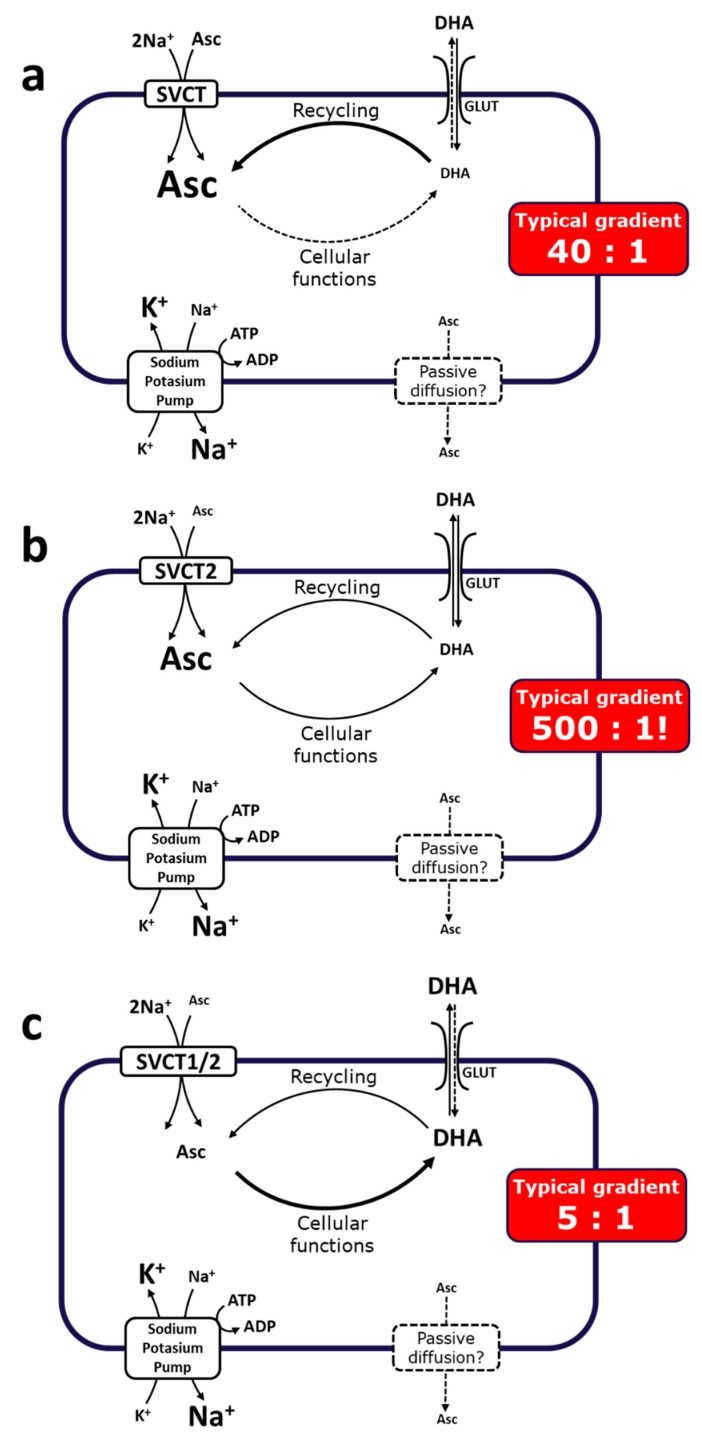
Tissue accumulation of vitC depends on both local and systemic conditions. The ratios are based on data obtained from guinea pigs that like human cannot synthesize vitC [7]. (**a**): During sufficiency, tissues accumulate vitC primarily through the sodium-dependent vitC transporters (SVCTs) perhaps with a small contribution from influx of DHA, which is rapidly converted to ASC. (**b**): During deficiency, prioritized retainment of vitC occurs in, for example the brain, at the expense of other tissues (**c**): where increased oxidative stress may result in elevated DHA concentrations, limited recycling capacity and poor tissue accumulation through DHA influx.

Apart from its remarkably high steady state concentration, the brain also distinguishes itself by being exceptional in the retention of vitC during states of deficiency [54,58,59,60,61,62,63,64]. This retention occurs at the expense of the other organs and has been proposed to be essential for the maintenance of proper brain function [63,65,66] (Figure 3). Also, during repletion, the brain, as well as the adrenal glands, has a remarkable affinity for ASC, and detailed in vivo studies in guinea pigs, which, like humans, are unable to synthesize vitC, have revealed that these tissues in particular are the fastest to re-establish homeostasis [7]. 

The mechanism(s) underlying the highly differential steady state concentrations of vitC in various tissues remains largely unknown. The potential existence of multiple tissue-specific isoforms of the SVCT2 has not been confirmed, leading to the assumption that the individual SVCT2 expression level of the cells of the tissues may define organ steady state levels of vitC subject to plasma availability. This implies that tissue and cell type composition are mainly responsible. However, in the brain of guinea pigs, for example, substantial differences in vitC steady state levels have been observed between the individual regions, with the highest concentrations being found in the cerebellum, which also appears to saturate first [7]. This does not directly coincide with cerebellum being the most neuron-rich brain region, although neurons contain the highest concentrations of vitC of the brains cells. Moreover, regional SVCT2-abundance has mostly been investigated through RNA expression levels leaving little information on the possible influence of, e.g., post-translational modifications, activation, and/or relocation of the functional protein to the cell membrane. 

#### 2.1.3. Metabolism

**Figure 4:**
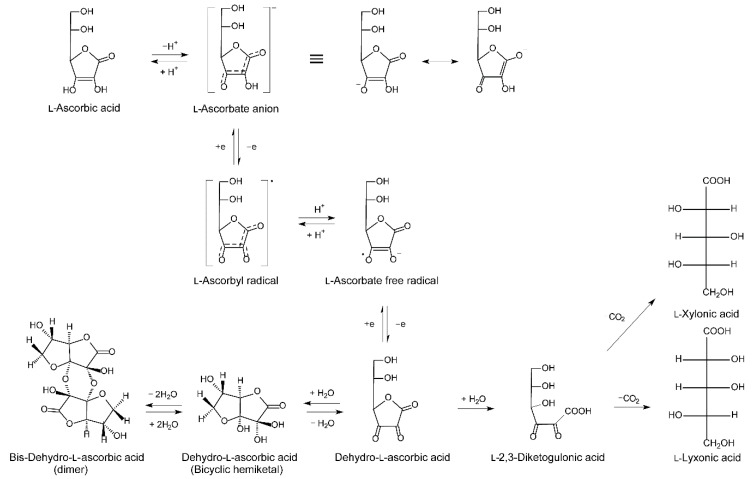
Schematic outline of vitC metabolism. Modified from [21].

In contrast to plants, where a number of ASC derivatives and analogues, including several glucosides, have been identified, only ASC exists in mammals [67]. The metabolism of ASC is intimately linked to its antioxidant function. Through its enediol structure (Figure 4) that is highly resonance stabilized and influenced by the acidity of the molecule, ASC serves as an efficient electron donor in biological reactions. In supplying reducing equivalents as either a cofactor or free radical quencher, ASC itself is oxidized to the comparatively stable radical intermediate, ascorbyl free radical, two molecules of which may be disproportionate at a physiological pH to one molecule of ASC and one of DHA [21,68]. As mentioned earlier, DHA is efficiently reduced intracellularly by a number of cell types, thereby preserving the ASC pool. Turnover of vitC is therefore particularly linked to the catabolism of DHA which occurs through hydrolysis to 2,3-diketogulonic acid and decarboxylation to l-xylonate and l-lyxonate, both of which can enter the pentose phosphate pathway for further degradation (Figure 4) [69]. 

#### 2.1.4. Excretion and Reuptake

**Figure 5:**
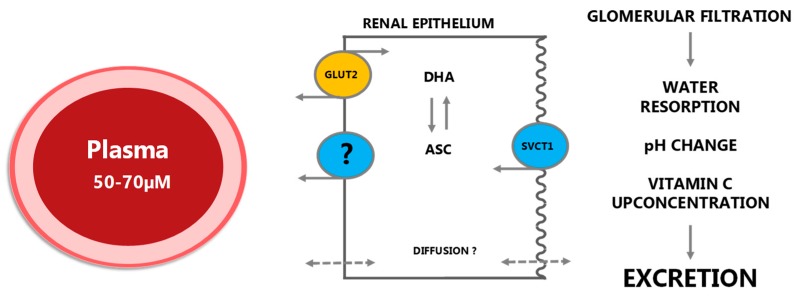
In the kidney, vitC is efficiently filtered by glomerulus to the renal tubule lumen. Reabsorption under vitC deficient conditions is primarily achieved by SVCT1 transporters in the apical membrane although diffusion from the luminal surface may also contribute to the overall uptake. As in the intestinal epithelium, ASC is presumably released to the blood stream through diffusion but the extent and mechanisms of this are not known in detail. GLUT2 transporters are located in the basolateral membrane enabling transport of DHA to plasma. Under saturated conditions, vitC is quantitatively excreted. Modified from [5].

As a highly hydrophilic low molecular weight compound, ASC would be expected to be efficiently excreted through the kidneys. Indeed, ASC is quantitatively filtered through glomerulus by means of the hydrostatic pressure gradient and concentrated in the pre-urine subsequently to the resorption of water (Figure 5). Here, the pH drops to about five, resulting in an increased proportion of unionized ascorbic acid to that of ASC. The ascorbic acid increase from <0.01% in plasma to about 15% in the pre-urine, representing a concentration gradient of 1500:1, would for most molecules result in substantial passive reabsorption but does apparently not occur for ascorbic acid presumably due to its low lipid solubility. Instead, reuptake of ASC in the proximal renal tubules is controlled by saturable active transport through SVCT1. However, for individuals with saturated plasma levels, excretion of surplus vitC is quantitative [70,71]. 

The importance of SVCT1 for intestinal vitC uptake and, in particular, for renal reuptake has been illustrated by Corpe et al. who showed that *Slc23a1-/-* mice display an 18-fold increased excretion of ASC, lower body pool and vitC homeostasis, and increased mortality [32]. They also modelled the effect of known human polymorphisms in the SVCT1 on the plasma saturation level and came to the astonishing conclusion that the most severely affected SNP (A772G rs35817838) would result in a maximal plasma concentration of less than 20 µM [32], i.e., a potential life-long state of vitC deficiency regardless of intake. The renal reuptake of ASC is highly concentration-dependent. Levine and coworkers have shown in detail that the renal excretion coefficient of ASC ranges from 0 to 1 depending on the individual’s vitC status, i.e., corresponding to quantitative reuptake in individuals with poor vitC status and quantitative excretion in individuals with saturated status [70,71]. The fact that the excretion ratio is about 1 for intakes higher than about 500 mg/day in healthy individuals supports that passive reabsorption of vitC does not play a significant role in the kidneys.

#### 2.1.5. Steady State Homeostasis of Vitamin C Following Oral Administration/Intake

Most low molecular weight drug pharmacokinetics can be modelled by first order kinetics within their therapeutic range, i.e., a doubling of the dose results in a doubling of the steady state plasma concentration. However, the dominant role of the saturable active transport mechanisms in the absorption, distribution, and excretion of ASC results in nonlinear dose-dependent pharmacokinetics. With increasing vitC intake, the plasma steady state concentration reaches a maximal level of about 70–80 µM [70,71]. From the available literature, it appears that a daily intake of about 200–400 mg of vitC ensures saturation of the blood in healthy individuals [20]. During periods of altered distribution due to temporary physiological needs such as pregnancy or increased turnover during disease or smoking, higher intakes are needed to maintain sufficient levels. 

It may be possible to exceed the homeostatic saturation level of 70–80 µM by several fold through multiple daily gram doses of vitC. At supraphysiological levels, vitC gradually adheres to first order kinetics as discussed under intravenous administration. Hence, it is possible to estimate that, for example, a dose of 2 g of vitC given three times a day is likely to result in a steady state plasma concentration of about 250 µM (calculations based to data from ref [13]). However, the possible health benefits from such supraphysiological levels have yet to be documented.

#### 2.1.6. Effect of Dosing Forms and Formulations

Several attempts have been made to bypass the maximum steady state plasma concentration of about 70–80 µM achievable through oral administration. A slow release formulation would theoretically extend the uptake period resulting in a prolonged and thus increased accumulated uptake thereby increasing the overall exposure. However, Viscovich et al. did not find any significant differences in exposure or other pharmacokinetic variables between plain and slow release vitC supplements given to smokers, neither at study start nor after 4 weeks of supplementation [29]. Another approach to increase the maximum achievable plasma concentration through oral administration has been liposomes. The pharmacokinetic properties of a bolus of four grams of liposome-encapsulated vitC were compared to those of plain vitC and placebo in eleven volunteers in a crossover trial [72]. The authors found a 35% increase in exposure (AUC0–4hours) with a plasma C_max_ of about 200 µM after 3 h. Unfortunately, plasma concentrations were not measured beyond the 4 h time point. In an attempt to show a potential biological significance of increased plasma vitC status, the participants were subjected to a 20-min partial ischemia induced by a blood pressure cuff at 200 mm Hg. However, no beneficial effect on ischemia-reperfusion-induced oxidative stress was observed on lipid peroxidation over that of the non-encapsulated dose of vitC [72]. Regardless, this technology has shown some promise and continues to be explored in anticancer therapy, where chemotherapeutics can be delivered together with vitC for a potentially synergistic effect [73]. In another sophisticated approach, the particular ability of the brain to take up vitC has been used by linking ASC to the surface of liposomes containing chemotherapeutics thereby making a brain-specific drug delivery system by using the endogenous vitC transport mechanisms [74]. 

### 2.2. Intravenous Route of Administration

**Figure 6:**
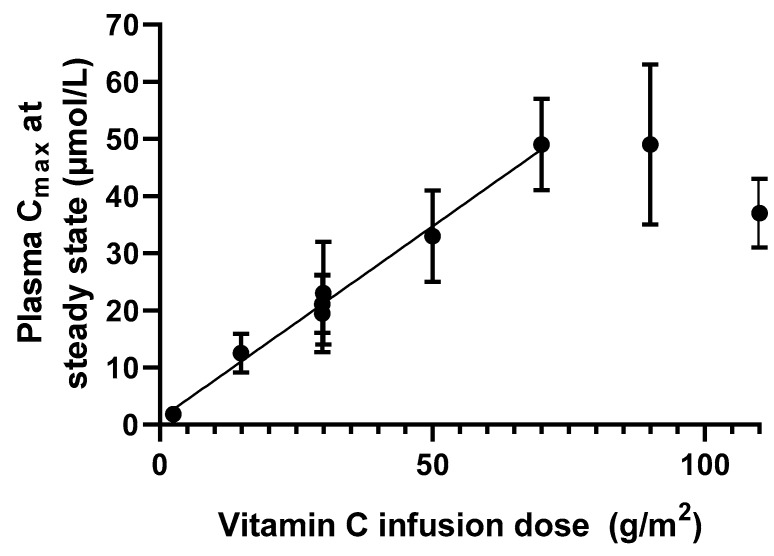
Relationship between infusion dose of vitC and plasma C_max_ in cancer patients as compiled from [13,75]. The data suggests that a linear relationship between dose and C_max_ exists for doses between 1 and 70 g/m^2^ (*p* < 0.001, *r*^2^ > 0.99), while higher doses results do not translate into higher plasma C_max_.

Intravenous administration of drugs generally produces a predictable plasma concentration by avoiding absorption limitations, resulting in 100% bioavailability. For vitC specifically, intravenous administration bypasses the saturable absorption mechanisms. This virtually removes the upper limit of the maximum achievable plasma concentration. Parenteral administration of vitC is typically handled by intravenous infusion. This approach results in a predictable plasma steady state concentration that will remain constant until infusion is discontinued. For vitC, a linear relationship between dose and C_max_ can be observed for doses up to about 70 g/m^2^ in humans as complied from clinical pharmacokinetic studies, resulting in a plasma concentration of about 50 mM (Figure 6, calculations based on [13,75]). For higher doses, the linearity seems to disappear and resembles a level of saturation. However, more data are needed to establish if 50 mM constitutes an upper steady state vitC concentration in plasma.

#### 2.2.1. Distribution

As for all compounds in circulation, the distribution of vitC following infusion depends at least initially on the vascularization of the various tissues. Whereas the millimolar plasma concentrations do not seem to affect normal tissue distribution beyond saturation, particular interest has been devoted the poorly vascularized tumors as ASC has shown to be cytotoxic to cancer cells but not normal cells at high concentrations in in vitro and in vivo studies, possibly through a pro-oxidant function [76,77,78]. Campell et al. [79] measured ASC concentrations in tumor tissue following high-dose vitC administration in a mouse model and found that daily injections were necessary to delay tumor growth and suppress the transcription factor hypoxia-inducible factor 1. Interestingly, it was also found that elimination was significantly delayed in tumor compared to normal tissue [79], which may help in preserving the effect of ASC in tumors between infusions. In an attempt to mimic tissue diffusion rates and availability in both normal and tumor tissue, Kuiper and coworkers [80] used a multicell-layered, three-dimensional pharmacokinetic model to measure ASC diffusion and transport parameters through dense tissue in vitro. They were able to simulate diffusion under a number of conditions, including tumors, and concluded that supraphysiological concentrations of ASC, achievable only by intravenous infusion, are necessary for effective delivery of ASC into poorly vascularized tumors [80]. Using these data, it was recently rationalized that normal body saturation obtained by adequate oral dosing will be able to diffuse to cover the distance between vessels in normal well-perfused tissue, and thus provide sufficient vitC for the entire body. In contrast, this diffusion distance is insufficient to increase the vitC content of tumors with poor vascularization, which requires above millimolar concentrations plasma concentrations for effective vitC diffusion [81]. Other than that, very little is known about the organ and tissue homeostasis following intravenous infusion of high-dose vitC.

#### 2.2.2. Metabolism and Excretion

In normal tissue, metabolism of ASC has not been shown to deviate from the general pattern illustrated in Figure 4. However, in poorly vascularized tumor tissues, high-dose vitC combined with the hypoxic tumor environment has been proposed to promote the formation of cytotoxic levels of hydrogen peroxide, thus providing a putative mode of action and a potential role of ASC in cancer treatment [26,82,83].

Following high-dose intravenous administration of vitC, the dose-dependency of the elimination phase, as evident at levels below saturation as described above, is surpassed [84]. VitC is quickly eliminated through glomerular filtration with no significant reuptake. This renders the half-life constant and the elimination kinetics first order [13]. Several pharmacokinetic studies of high-dose vitC have calculated a constant elimination half-life of about 2 h following the discontinuation of intravenous infusion [13,75,85]. This suggests that the millimolar plasma concentrations achieved by intravenous infusion are normalized to physiological levels in about 16 h. In this perspective, the observation that tumor tissue may maintain an elevated level for as much as 48 h is interesting [79], and may be mediated by increased stability in the hypoxic tumor environment, but most likely also by the delayed clearance due to poor vascularization. 

## 3. Factors Affecting Vitamin C Homeostasis and Requirements

As described in detail in the above, vitC homeostasis is tightly controlled in healthy individuals giving rise to a complex relationship between the steady state levels of the various bodily organs and tissues. This interrelationship depends primarily on the availability of vitC in the diet and the specific “configurations” and expression levels of SVCTs of the tissues. However, a number of other factors may interfere with the body’s attempt control the vitC homeostasis, and some major contributors are discussed below.

### 3.1. Influence of Polymorphisms 

With the acknowledgement of the importance of SVCTs for regulation of vitC homeostasis and the evolution of genomic sequencing techniques, it has become clear that a large number of polymorphisms exist that influence the steady state level of vitC. This has been reviewed in detail elsewhere [3], but little is known about the potential clinical impact of these. A Mendelian randomization study in 83,256 individuals from the Copenhagen General Population Study used a genetic variant rs33972313 in Slc23a1 resulting in higher than average vitC status to test if improved vitC status is associated with low risk of ischemic heart disease and all-cause mortality [86]. The authors found that high intake of fruits and vegetables was associated with low risk of ischemic heart disease and all-cause mortality. Effect sizes were comparable for vitC, albeit not significantly. As mentioned earlier, modelling studies have proposed that the functionally poorest SVCT allele identified so far (A772G, rs35817838) results in a plasma saturation level of only one fourth of that of the background population corresponding to a condition of life-long vitC deficiency [9]. It would indeed be interesting to test how this allele compares for morbidity and mortality.

### 3.2. Smoking

Smoking is a major source of oxidants and estimates have suggested that every puff of a cigarette equals the inhalation of about 1014 tar phase radicals and 1015 gas phase radicals [87]. Not surprisingly, this draws a major toll on the antioxidant defense of the body as demonstrated by a persistent association between tobacco smoke and poor antioxidant status in general, and poor vitC status in particular [17,25]. Active smoking typically depletes the vitC pool by 25–50% compared to never-smokers [88], while environmental tobacco smoke exposure results in a drop of about half that size [89,90]. The direct cause of the smoking-induced vitC depletion has been investigated, and smoking cessation has been shown to immediately restore about half of the vitC depletion observed as a result of smoking [91]. This immediate albeit partial recovery has pointed towards an oxidative stress mediated depletion of vitC caused by smoking. Moreover, both oxidative stress and ASC recycling are induced by smoking regardless of antioxidant intake [18,92]. However, the lack of full recovery suggests that other factors also contribute to the lower vitC status among smokers. Studies have suggested that the difference in vitC status between smokers and nonsmokers is not related to altered pharmacokinetics of vitC [28,29]. However, as smokers in general have a lower intake of fruits and vegetables and a larger intake of fat compared to nonsmokers [93], this may account for the difference in vitC levels observed between ex-smokers and never-smokers [19]. Indeed, an analysis of the Second National Health and Nutrition Examination Survey (NHANES II) confirmed that the vitC intake of smokers is significantly lower than that of nonsmokers, but also that the increased risk of poor vitC status was independent of this lower intake [94]. 

Various attempts have been made to estimate the amount of vitC needed to compensate for tobacco smoking. Schectman et al. analyzed the NHANES II data, comparing daily intake vs. serum concentrations of vitC among 4182 smokers and 7020 non-smokers. They estimated by regression analysis that smokers would need an additional 130 mg/day to overcome the adverse effect of smoking on vitC status [95]. In a separate analysis, it was concluded that smokers need an intake > 200 mg/day to lower the risk of vitC deficiency to that of nonsmokers [96]. These results were later indirectly supported by Lykkesfeldt et al. using a different approach. Measuring the steady state oxidation ratio of vitC in smokers and nonsmokers, it was shown that in particular smokers with poor vitC status had an increased steady state oxidation of their vitC pool compared to nonsmokers [17]. The authors concluded that smokers need at least 200 mg vitC per day to compensate for the effect of smoking on the oxidation of vitC [17]. These data stand in contrast to previous data by Kallner et al., who used ^14^C-labelled ASC to estimate the turnover of vitC in smokers [97]. Seventeen male smoking volunteers between 21 and 69 years of age and weighing between 55 and 110 kg received doses from 30 to 180 mg/day and were instructed to ingest a diet completely devoid of vitC. Urinary excretion of radioactivity was used to estimate the vitC pharmacokinetics using a three-compartment model. Based on these data, Kallner et al. concluded that smokers needed only about 35 mg more than nonsmokers per day to compensate for their habit [97]. This recommendation was later adopted by the Institute of Medicine in their dietary reference intakes [22]. However, several problems are associated with the latter study. Namely, radioactivity rather than ASC per se was quantified as a surrogate for vitC excretion. Moreover, only 17 individuals with considerable variation in age and body composition were included in the study. Finally, these studies were carried out prior to the identification of the SVCTs and their importance for the nonlinear pharmacokinetics of vitC at physiological levels. In fact, such a dose-concentration relationship formally rules out the use of compartment as well as noncompartment kinetic modelling, as the fundamental assumption of a terminal first order elimination phase is not fulfilled. Thus, it appears likely that the turnover in smokers may be underestimated by Kallner et al. 

### 3.3. Pregnancy

Several preclinical studies have illustrated the importance of vitC in early development, in particular that of the brain and cognition [60,63,98,99,100]. In humans, studies have shown that poor maternal vitC status results in increased fetal oxidative stress, impaired implantation and increased risk of complications including preeclampsia [101,102]. It is not clear to what extent vitC supplementation may ameliorate this risk. The few controlled studies that have been carried out have produced mixed results [103,104,105,106], but unfortunately, none of them have considered vitC status in the recruitment or group allocation process and they are therefore of limited value. 

During pregnancy, the human fetus relies completely on an adequate maternal vitC intake and transplacental transport of vitC. Experimental evidence suggests that this transport is primarily governed by SVCT2 and thus constitutes the primary means of fetal vitC supply [60]. Expectedly, maternal vitC status has been shown to gradually decline from the 1^st^ to 3^rd^ trimester, a change not only explainable by the increased volume of distribution but rather by the selective accumulation across the placenta [107]. Fetal and postnatal steady state concentrations exceed those of the mother, and both during pregnancy and lactation, most authorities recommend an increased intake ranging from 10 to 35 additional mg vitC/day to compensate for this increased draw on maternal resources [22]. 

### 3.4. Disease

A plethora of disease conditions, including infectious diseases, cancer, cardiovascular disease, stroke, diabetes, and sepsis, have been associated with poor vitC status (reviewed in [19,20]). Considerable epidemiological evidence has shown vitC deficiency to negatively affect independent risk factors of, for example, cardiovascular disease development [14]. However, causal linkage between disease etiology and vitC status remains scarce, except for that of scurvy [108]. The decreased vitC status in disease is often explained by a combination of a sometimes massively increased turnover due to oxidative stress and inflammation and a decreased dietary intake of vitC associated with the disease [81,109].

An obvious display of increased vitC turnover in critical illness is that large doses are often needed to replete the individual to the level of a healthy control. These doses exceed those necessary to saturate a healthy individual by many-fold [110]. One current example is sepsis patients where systemic inflammation and oxidative stress presumably increases the expenditure of vitC [111,112]. Recently published data on critically ill patients (*n* = 44, both septic and nonseptic patients) show that actual plasma vitC concentrations are on average 60% lower than the values predicted from patient vitC intake during hospitalization (either enteral or parenterally administered nutrition) [110]. Although several causes of the apparent vitC depletion are likely, e.g., interactions with administered care and therapeutics potentially affecting vitC bioavailabilty, the data suggest significant alterations in the pharmacokinetics of vitC in this group of patients, reflected by the discrepancy in the almost linear course of the plasma concentration curve opposed to the predicted increase over time. Whether reestablishing normal vitC status in critically ill patients has a significant clinical impact on disease prognosis remains to be established, but promising results are emerging [113,114] and controlled trials are under way. A very recent meta-analysis suggests that vitC therapy significantly shortens the stay of patients in the intensive care unit [115].

In diabetes, reduced levels of plasma vitC is reported in both insulin demanding and noninsulin demanding diabetic patients [116,117,118,119]. A prospective evaluation of older adults in the National Institutes of Health-American Association of Retired Persons (NIH-AARP) Diet and Health Study cohort, indicate that the use of vitC supplementation may reduce the risk of diabetes, supporting further investigations and controlled trials to identify a putative relationship between vitC levels and diabetes [120]. Supplementation with vitC (500 mg/day) increased insulin sensitivity and the expression of the SVCT2 transporter in skeletal muscle in type 2 diabetic patients [121], supporting findings that an intake of high-dose ascorbic acid (above 1 mg/day) exerted a beneficial effect on maintaining blood sugar homeostasis and decreasing insulin resistance in type 2 diabetic patients [119]. In a randomized controlled cross-over study of type 2 diabetes patients, an intake of 500 mg vitC twice daily for four months significantly improved glucose homeostasis as well as decreased blood pressure compared to placebo treated controls, linking vitC supplement to improved blood-sugar balance and cardiovascular function [122]. Positive effects of vitC on vascular hallmarks linked to diabetes have previously been indicated; in young diabetes type 1 patients, poor vitC status was linked to increases in the arterial vascular wall, indicating a putatively increased risk of atherosclerotic disease in these patients [123]. In type 2 diabetic patients with coronary artery disease, a high-dose supplementation (2 mg/day) for 4 weeks reduced circulating markers of thrombosis, supporting a beneficial role of vitC on the vascular system [124]. Collectively, the above evidence suggests that a higher metabolic turnover of vitC in diabetes can be counter-balanced by supplementation. However, if it also improves the long-term prognosis remains to be evaluated.

## 4. Concluding Remarks

The pharmacokinetics of vitC is complex, dose-dependent, and compartmentalized at physiological levels, while independent of dose and first order at pharmacological levels. The lack of this fundamental knowledge has left deep traces of design flaws, misconceptions, misinterpretations, and erroneous conclusions in the scientific literature. Unfortunately, these inherited problems continue to hamper our ability to properly evaluate the role of vitC in human health and its potential relevance in disease prevention and treatment. So far, the overtly exaggerated optimistic view that enough vitC can cure everything has been battling the dismissive negligence of refusal to re-examine the literature based on new evidence. The balance between these two extremes needs to be identified in order to realize the potential of vitC in both health and disease for the future.
